# Numerical Simulation and Experimental Study the Effects of Process Parameters on Filament Morphology and Mechanical Properties of FDM 3D Printed PLA/GNPs Nanocomposite

**DOI:** 10.3390/polym14153081

**Published:** 2022-07-29

**Authors:** Mingju Lei, Qinghua Wei, Mingyang Li, Juan Zhang, Rongbin Yang, Yanen Wang

**Affiliations:** 1 Industry Engineering Department, School of Mechanical Engineering, Northwestern Polytechnical University, Xi’an 710072, China; leimingju0852@126.com (M.L.); lmy9366@gmail.com (M.L.); juanzhang@mail.nwpu.edu.cn (J.Z.); yang2021@mail.nwpu.edu.cn (R.Y.); 2 Xinjiang Institute of Engineering, School of Mechatronic Engineering, Urumchi 830000, China; 3 Institute of Medical Research, Northwestern Polytechnical University, Xi’an 710072, China

**Keywords:** fused deposition modeling, PLA/GNPs nanocomposite, filament morphology, mechanical property, numerical simulations, experimental validation

## Abstract

The selection of optimal process parameters has a decisive effect on the quality of 3D printing. In this work, the numerical and experimental methods were employed to investigate the FDM printing deposition process of PLA/GNPs nanocomposite. The effect of process parameters on cross-sectional morphology and dimension of the deposited filament, as well as the mechanical property of the FDM printed specimens were studied. The extrusion and the deposition process of the molten PLA/GNPs nanocomposite was simulated as a fluid flow by the paradigm of CFD, the effects of printing temperature and shear rate on thermal-physical properties, such as viscosity and surface tension, were considered in models. Under the assumptions of non-Newtonian fluid and creep laminar flow, the deposition flow was controlled by two key parameters: the nozzle temperature and the nozzle velocity. The numerical model was verified by experiments from four aspects of thickness, width, area, and compactness of the deposited PLA/GNPs nanocomposite filament cross-section. Both the numerical simulation and experiment results show that with the increase of nozzle temperature and nozzle velocity, the thickness, area, and compactness of the deposited filament decreases. While the width of deposited filament increased with the increase of nozzle temperature and decrease of nozzle velocity. The decrease in thickness and the increase in width caused by the change of process parameters reached 10.5% and 24.7%, respectively. The tensile strength of the printed PLA/GNPs specimen was about 61.8 MPa under the higher nozzle temperatures and velocity condition, an improvement of 18.6% compared to specimen with the tensile strength of 52.1 MPa under the lower nozzle temperatures and velocity condition. In addition, the experimental results indicated that under the low nozzle velocity and nozzle temperature condition, dimensional standard deviation of the printed specimens decreased by 52.2%, 62.7%, and 68.3% in X, Y, and Z direction, respectively.

## 1. Introduction

Fused deposition modeling (FDM), also known as extrusion-based three-dimensional (3D) printing, is a manufacturing technique that utilizes thermoplastic material to create models, prototypes, and even end-products [1]. The basic principle of the FDM technology is the hot-melt extrusion and layer deposition process of the filament through the nozzle of the extruder according to the generated pattern and G-codes sent to machine by the software [2]. This technology builds the products in layers from the bottom to the top; the thermoplastic feedstock is heated into semiliquid state in the head of the printer containing a heated liquefier, extruded through the nozzle, and deposited layer by layer onto the building substrate. Driven by the thermal diffusion process, the newly deposited filament is welded to the previous layer, solidified, and attached to the previous layer after cooling. This procedure is repeated for each layer until the desired final object is completed [3]. The detail review of the FDM techniques and its latest advances are provided in [4].

Compared with other mature additive manufacturing (AM) technologies, FDM technology is one of the most representative technologies in AM, being able to neatly and safely manufacture products with complex geometries and reasonable dimensional accuracy in the office-friendly environment [5]. FDM provides a simple fabrication process and cost-effective method to process a variety of thermoplastic polymers and their nanocomposites, such as PLA, ABS, PLGA, PA, and PET, using low-melting-point filament feedstock [6]. Moreover, FDM printer is more and more popular and widely accepted in industry for its advantages of small size, easy to use, universal operation interface, and capability of manufacturing multi-functional parts. With the current advances in process, new materials, and machine and part quality [7], FDM technology is rapidly maturing and has, obviously, shown unlimited application potential in different fields, such as automotive [8], aerospace [9], and medical care [10].

Despite the apparent advantages shown by FDM technique, some practical drawbacks and limitations encountered in the final specimen hinder its further development and industrial application. One major issue is that the parts manufactured by FDM always show unavoidable porosity formation and weak interface bond compared to the conventional compression and injection molding methods, resulting in poor mechanical properties, uncontrollable dimensional accuracy and surface quality, and even defects of gap and warpage. To address these problems, researchers have proposed various approaches and conducted corresponding tests. These studies mainly include two aspects: one is to focus on the modification research of the feedstock used in FDM. Particle, fiber, and nanomaterial reinforcements such as iron or copper particles [11], inorganic salt particle [12], glass fibers [13], carbon fibers [14], carbon nanofiber [15], and montmorillonite [16] are introduced into the thermoplastic polymer to improve the mechanical properties of FDM printed parts by enhancing the cross-linking state and crystallinity of chains. Due to the unique biocompatibility and electrical and thermal properties of the reinforcements, the functional composite modification of FDM feedstock endows the FDM printed parts to have high thermal stability, electrical conductivity, biocompatibility, and electromagnetic shielding. In our previous work, the improved mechanical properties, thermal stability and cell viability of the FDM PLA/GNPs nanocomposite samples have been successfully fabricated by adding biocompatibility GNPs into PLA [17].

In addition, the process parameters are found to be another key factor that highly determine the mechanical properties of FDM printed parts. Therefore, some researchers have focused on optimizing process parameters, such as orientation [18], infill density [19], layer height [20], raster angle [21], printing velocity [22], and extrusion temperature [23,24], to improve interface bonding property [25], mechanical strength [26], and dimensional accuracy [27] of the FDM printed parts at a lower cost. These studies try to establish empirical relationships between materials and process parameters with mechanical performances of the FDM printed parts, and to identify the key process parameters and their optimal combinations by using different statistical and heuristic methods [28]. The modification of feedstock and optimization process parameters of FDM printing are of great significance for improving mechanical properties, and expanding or enriching the applications of FDM printed parts. However, as the basic substance and structure foundation that consisting a FDM 3D printed component, limited research has been done on the (numerical) study of the flow and solidification process of the filaments. Furthermore, there are few studies on the prediction of the cross-sectional morphology and dimension of the FDM filament under different process parameters and their effect on the mechanical property of FDM printed parts. It is of great importance to investigate the morphology of FDM filament during the deposition process. Therefore, the numerical simulation of FDM deposition process and the effect of process parameters on filament morphology and mechanical properties of FDM printed parts are helpful to develop high strength and accuracy of FDM results.

According to the literature, the morphology and dimension of the FDM filament can be studied by experiments or computation [29]. The numerical simulation of the FDM process and its effects on filament morphology and mechanical properties of FDM printed components are difficult, because the deposition process of FDM filament is complex and contains multiple physical phenomena, which occur at different spatio-temporal scales. The initial numerical modeling efforts focuses on the thermal and thermo-mechanical properties of the printed components after the extrusion of the feedstock [30]. Following, the non-isothermal fluid flow and fabrication process of ceramic filament during FDM process are computational studied in a two-dimensional geometry as presented in [31]. Later, more 2D [32] and 3D [33] thermal models coupled with sintering and healing models [34] have been developed to compute the local temperature history of the deposited filaments as well as the temperature-induced residual stresses [35] and deformations [36]. Recently, the prediction of bond formation [37] between adjacent filaments and the mesostructure [33] of FDM printed parts, as well, the study of heat transfer [38,39,40] and the morphology [41,42] of filaments after leaving the nozzle, have become research hotspots in numerical simulation of FDM process. For example, Xia et al. [40] proposed a full analytical simulation of the construction and heat transfer of FDM process based on finite volume/front-tracking method, and investigated the effects of nozzle temperature and filament spacing on the shape of the FDM object. Du et al. [41] used CFD simulations of non-isothermal flows of the improved FDM process with laser-assisted heating. The numerical model has been used to predict the thermal field and the area of contact of the adjacent filaments, and tensile test has been carried out to examine process parameters of nozzle speed, laser power, and the ways of laser heating on the tensile strength of the FDM part. Comminal et al. [42] have utilized a coupled level-set/volume-of-fluid method to predict the cross-sectional morphology of the filament and printing force on the substrate in FDM, where the process parameters and dimensions of the numerical model were normalized by the nozzle diameter. Serdeczny et al. [43] further investigated the effect of layer thickness, spacing of the filaments, and deposition configuration on the mesostructure of FDM representative volume element numerically and experimentally. However, the numerical models assumed that the feedstock was an isothermal Newtonian fluid that neglects heat transfer, which makes it different to calculate the viscosity change of the filament and the heat transfer between the filament and the surrounding air during FDM process. Therefore, the accurate numerical models were used to simulate the FDM process by considering the effects of process parameters on the thermal-physical properties, and the influences of the cross-sectional morphology and dimension of FDM filament on the mechanical properties of FDM printed specimens under different process parameters were investigated. Currently, PLA/GNPs nanocomposite have been widely used in FDM 3D printing technology due to their excellent mechanical properties, thermal stability, potential application as biomaterials, highly electrical conductivity, and highly efficient EMI shielding property [17,44]. However, there is little in the literature on the numerical study of the deposition and molding process of PLA/GNPs nanocomposite in FDM process. The work of this paper will offer a valuable reference for the further research and application of FDM 3D printing PLA/GNPs nanocomposite. In this study, PLA/GNPs nanocomposites were used as testing materials and the cross-sectional morphology and dimensions of FDM printed PLA/GNPs nanocomposite filaments under different process parameters were investigated by numerical simulation and experiment, and the effects of process parameters on mechanical properties of FDM printed specimens were analyzed.

## 2. Materials and Methods

### 2.1. Preparation of PLA/GNPs Nanocomposite

The preparation method of PLA/GNPs nanocomposites used in this paper is based on our previous work [17]. A solvent-based mixing method was used to prepare PLA/GNPs nanocomposites. Specifically, the PLA pellets with molecular weight of 100,000 was immersed in dichloromethane (DCM) of 150 mg/mL and stirred under 50 °C for 5 h. Then, the graphite nanoplatelets (GNPs) were dissolved in N-dimethylformamide (DFM) of 5 mg/mL under ultrasonic dispersion for 1 h. Then, we mixed two obtained solutions and stirred vigorously for 12 h. Finally, the solution mixture was precipitated in ethanol and dried in a vacuum vessel at 40 °C for 24 h to remove the solvent. The precipitates were extruded by a single-screw extruder and the filaments with a diameter of 1.75 mm were prepared for the 3D printing experiment. According to our previous research results, PLA/GNPs nanocomposite with 2 wt% GNPs possesses the optimal mechanical properties and biocompatibility, so here the nanocomposite was selected as the research material both in numerical simulation and the printing experiment.

### 2.2. Physical and Numerical Models

In the simulations of FDM printing PLA/GNPs nanocomposite presented here, we focus on the extrusion and deposition of molten PLA/GNPs nanocomposite from moving nozzle to fixed substrate, as well as the solidification process of deposited material in the region between the printing head and the substrate. The extrusion and deposition process of molten PLA/GNPs nanocomposite was simulated as a fluid flow by the paradigm of CFD. The molten PLA/GNPs nanocomposite and the surrounding air were modelled as immiscible two-phase flows in the Eulerian framework, where the first fluid phase represents the molten biomaterial and the second fluid phase represents the surrounding air. The primary phase of the molten PLA/GNPs nanocomposite was assumed as a non-Newtonian liquid and the surrounding air is an incompressible Newtonian fluid. Because the pressure of the molten PLA/GNPs is not too high and the surrounding air velocity is much lower during the FDM process, the material–air system can be treated as an incompressible fluid.

**Figure 1:**
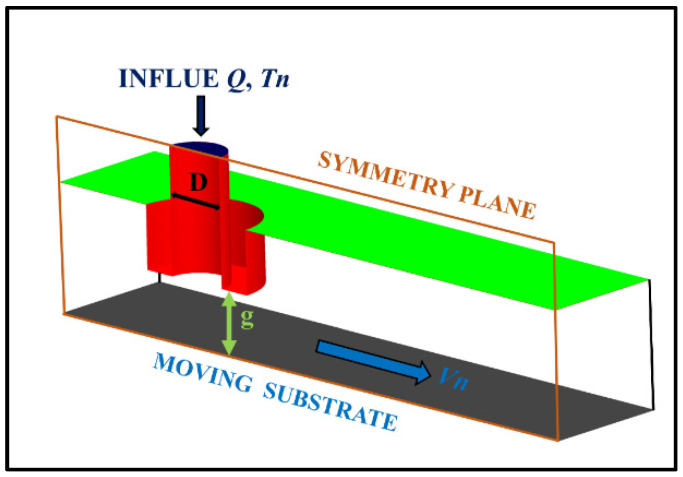
Three-dimensional geometry of the numerical model.

Three-dimensional geometry of computational model is given in Figure 1. The computational domain is a 1.2 mm × 2.4 mm × 4 mm hexagonal box containing the nozzle and the substrate as well as the gap between them. To simplify the computational model, the printing head was modelled as a no-wall thickness cylindrical tube with an inner diameter of 0.4 mm and length of 1.2 mm. The nozzle was treated as a no-slip diabatic wall which defined the temperature boundary. The top surface and side wall of the domain were outlet boundaries, which were open and allowed the extruded PLA/GNPs nanocomposite and the ambient cold air to freely move in and out of the domain. Considering that the movements of the printing head and the substrate are relative to each other in the actual FDM printing process, the opposite motion configuration of the substrate moving while the nozzle is fixed was adopted in the present model. The substrate was treated as a rigid, no-slip diabatic wall, allowing heat transfer between the substrate and the deposited material.

The effect of printing parameters on deposition quality was studied in detail by the numerical method in this paper. Nozzle velocity ($V_{n}$, defined by the substrate velocity boundary) and nozzle temperature ($T_{n}$, defined by the nozzle temperature boundary) were adopted as the two key control variables to characterize the computational model. The velocity of the substrate was set as 30, 40, and 50 mm/s, respectively. The temperature of the nozzle and the extruded material was set as 180, 200, and 220 °C. The initial temperature of the air was set as 25 °C, the feed rate of melt PLA/GNPs nanocomposite was fixed at 5.024 ${\mathrm{mm}}^{3}/s$, and the temperature of substrate was kept at 25 °C. The z-axis gap between the nozzle and the substrate is *g* (0.4 mm), which could be considered as the layer thickness. The printing parameters of the numerical model are given in Table 1.

Numerical simulation of the FDM printing process involves the flow of molten material, the free surface movements, an appropriate rheological model used to represent the viscosity of printing material, and heat transfer. Under the assumption of an incompressible two-phase flow including nanocomposite melt and surrounding air, the dynamic flow field is governed by the continuity equation, momentum equation, and energy equation, which can be expressed as:

(1) ∇·u=0,

(2) $$\frac{\partial \left(\rho \left(\emptyset \right)u\right)}{\partial t}+\nabla \cdot \left(\rho \left(\emptyset \right)uu\right)-\nabla \cdot \left(2\eta \left(\emptyset \right)D\right)=-\nabla p+H_{\varepsilon }\left(\emptyset \right)\nabla \cdot \left(2\left(\beta -1\right)\eta \left(\emptyset \right)D\right)+H_{\varepsilon }\left(\emptyset \right)\nabla \cdot \tau ,$$

(3) $$\frac{\partial \left(\rho \left(\emptyset \right)C_{\nu }\left(\emptyset \right)T\right)}{\partial t}+\nabla \cdot \left(\rho \left(\emptyset \right)C_{\nu }\left(\emptyset \right)uT\right)-\nabla \left(\kappa \left(\emptyset \right)\nabla T\right)=H_{\varepsilon }\left(\emptyset \right)\tau_{ij}:\nabla v_{i},$$

where

(4) $$D=\frac{1}{2}\left(\nabla u+\nabla u^{T}\right),$$

(5) $$H_{\varepsilon }(\emptyset )\;=\;\{\begin{array}{ll} 0 & \mathrm{if}\;\emptyset \;<-\varepsilon \\ \frac{1}{2}[1+\frac{\emptyset }{\varepsilon }+\frac{1}{\pi }\sin(\frac{\pi \emptyset }{ɛ})] & \mathrm{if}\;|\emptyset |\;\leq \varepsilon \\ 1 & \mathrm{if}\;\emptyset \;>\varepsilon \end{array}$$

where, u is the velocity field, η is the viscosity field, and ρ is the density including buoyancy effect caused by thermal expansion of air. T, ∁, and κ are temperature, specific heat capacity, and thermal conductivity, respectively. ∅ is a level set (LS) function identifying the interface between the molten PLA/GNPs nanocomposite and the air by calculating the normal and mean curvature of the interface. $D=\left(1/2\right)\left(\nabla u+\nabla u^{T}\right)$ is the rate of deformation tensor. H is the smoothed Heaviside function that ensure the controlling equations can be solved in the whole computational domain [45]. ε is a parameter related to the thickness of interface, in this work, ε=1.5Δx [46].

Due to different phases having different properties, the constant of the density and viscosity in each phase can be defined using the LS function and the sharp jump of interfacial fluid properties. The velocity and density are smoothed over a transition zone through the interface:

(6) $$\rho \left(\emptyset \right)=\rho_{a}+\left(\rho_{p}-\rho_{a}\right)H_{\varepsilon }\left(\emptyset \right),$$

(7) $$\eta \left(\emptyset \right)=\eta_{a}+\left(\eta_{p}-\eta_{a}\right)H_{\varepsilon }\left(\emptyset \right),$$

(8) $$C_{\nu }\left(\emptyset \right)=C_{a}+\left(C_{p}-C_{a}\right)H_{\varepsilon }\left(\emptyset \right),$$

(9) $$\kappa \left(\emptyset \right)=\kappa_{a}+\left(\kappa_{p}-\kappa_{a}\right)H_{\varepsilon }\left(\emptyset \right),$$

Here, the subscript *p* and *a* represent PLA/GNPs nanocomposite and air, respectively.

The free surface of two-phase flows including molten PLA/GNPs nanocomposite and surrounding air was reconstructed using the coupled level-set/volume-of-fluid method (CLSVOF). The CLSVOF method is an accurate interface reconstruction algorithm, in which the VOF function is used to determine the volume fraction of the liquid phase in each cell, and the level set function LS (a smooth function) is used to calculate the interface normal and mean curvature. In order to maintain the conservation of mass, the interface was constructed by using LS function modified from the VOF function. The coupling of LS and VOF methods takes place during the interface reconstruction. For more details about the CLSVOF method, refer to the articles in [47].

The heat transfer includes the conduction between hot PLA/GNPs nanocomposite and the substrate, as well as the convection between the deposited hot nanocomposite and its ambient air. Heat conduction between molten PLA/GNPs nanocomposite and substrate is expressed as $Q=k_{p}A\Delta T$, $k_{p}$ is the thermal conductivity of the nanocomposite. The thermal convection plays an important role in the cooling process of the molten liquid, the buoyancy effect caused by thermal expansion of air is calculated by introducing Boussinesq approximation into the gravity term of momentum equation.

To accurately simulate the deposition process of PLA/GNPs nanocomposites, an appropriate viscosity model is needed to characterize the rheological properties of the nanocomposites. In principle, the viscosity of thermoplastics in extruded additive manufacturing depends on temperature, shear rate, and pressure. Since the deposition process is non-isothermal and the temperature range of molten nanocomposites is large, the effect of temperature change on the deposition flow cannot be ignored. Once the molten nanocomposite flows out of the nozzle, the hot nanocomposite will undergo a glass to liquid transition. However, in the small gap between the extruder outlet and the moving substrate, the pressure is close to the ambient pressure after deposition, so we assume that the variations of pressure have a negligible effect on the deposition flow. The pressure changes little when the molten nanocomposite flows out of the extruder orifice. Thus, the effect of temperature and shear rate on cooling and solidification phenomena of the FDM process were considered in present computational models. The Cross-WLF, which is the most appropriate model to study both filling and packing phases, is applied to describe the viscosity change of the molten material with temperature and shear rate.

The expression of the Cross-WLF model is as follows:

(10) $$\eta_{m}\left(T,\dot{\gamma }\right)=\frac{\eta_{0}}{1+{\left(\eta_{0}\dot{\gamma }/\tau^{*}\right)}^{1-n}}$$

where, $\dot{\gamma }$ is the shear strain rate, calculated by $\dot{\gamma }=\sqrt{2D:D}$. $\tau^{*}$ and *n* are the critical stress at the transition to shear thinning and the non-Newtonian index of the Cross-WLF model, respectively. $\eta_{0}$ is the melt viscosity at zero-shear-rate, a function of temperature and pressure, which is expressed as:

(11) $$\eta_{0}=D_{1}exp\left(\frac{-A_{1}\left(T-T^{*}\right)}{A_{2}+D_{3}p+\left(T-T^{*}\right)}\right)$$

where, $T^{*}=D_{2}+D_{3}\cdot P$ is the glass transition temperature of the melt which depends on the pressure. $A_{1}$ is the model constant, which represents the temperature dependence of the glass transition temperature of the melt at zero shear rate. $A_{2}={\overset{`}{A}}_{2}+D_{3}\cdot P$ is the model parameter, which is determined by the type of nanocomposite melt. $D_{1}$ is the model constant for recording melt viscosity at zero shear rate, glass transition temperature, and atmospheric pressure. $D_{2}$ is the model constant for recording the glass transition temperature. $D_{3}$ is the model constant, which represents the change of glass transition temperature of the melt with pressure. In this work, the viscosity function was used for PLA/GNPs nanocomposite, the parameters in Cross-WLF model were given as: $A_{1}=20.194$,$\;A_{2}=51.6$, $D_{1}=3.31719\times 10^{+9}$, $D_{2}=100$. As the pressure difference inside the nanocomposite has little effect on the viscosity, the pressure effect can be ignored and $D_{3}=0$. Considering that the viscosity value is very high at low temperature and low shear rate, 8000 Pas is taken as the maximum viscosity value, which is large enough to avoid the model yielding an excessive viscosity value at a low temperature and shear rate while ensuring the solidification of the injected material. The material properties and variables are summarized in Table 2.

### 2.3. Principle of the Numerical Algorithm

**Figure 2:**
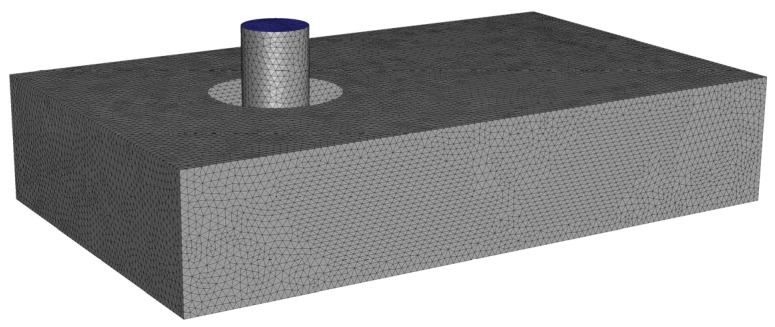
Details of cut-cell mesh in simulations.

The fluid flow was simulated by using the general finite element analysis software ANSYS Fluent R21. The governing equations were solved by a numerical method introduced in reference [47]. The computational domains were mashed with a non-staggered Cartesian cut-cell grid to form a series of nonoverlapping control volumes based on the finite-volume method. The governing equations were discretized on grid to form a control volume, and all discrete components were stored on the same nodes of the grid. A set of discrete equations were obtained by integrating the governing equations to be solved over each control volume. Following the grid center discretized, the numerical solver calculates discrete values of the continuous velocity and pressure fields in the center of the control volumes. The discrete unknowns between the nodes of the control volume were interpolated from the discrete values on the nodes according to the central difference scheme. The maximum size of the control volumes was set as 0.02 mm, the time-step interval was set to 0.01 s. The details cut-cell mesh used in the simulations were represented in Figure 2.

**Figure 3:**
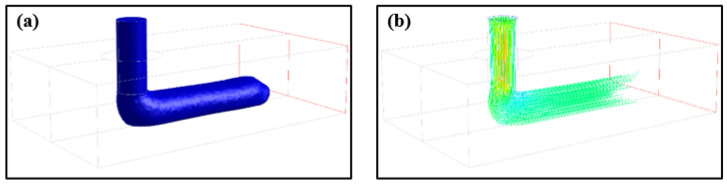
Simulation result under process condition of $V_{n}$ = 40 mm/s and $T_{n}$ = 200 °C: (**a**) Free surface of the deposited PLA/GNPs nanocomposite filament; and (**b**) streamlines of the deposited PLA/GNPs nanocomposite melt.

The free surface was reconstructed using an algebraic coupled level-set/volume-of-fluid method (CLSVOF) advection algorithm, where the position of surface was tracked by calculating the transport of two additional field variables. The VOF advection algorithm was adopted to reduce the excessive numerical diffusion caused by discontinuous changes of VOF function. The fractional step method or operator-split method was introduced to the VOF advection algorithm to simplify the solving of VOF function. The interface reconstruction was made through LS advection algorithm by evaluating the normal of interface. The LS advection function was discretized by fifth-order essentially nonoscillatory (ENO) scheme in space and third-order total variation diminishing (TVD) Runge–Kutta scheme in time. The continuous velocity and pressure fields of the control volumes were calculated through the velocity–pressure decoupling method based on semi-implicit pressure-linked equation algorithm (SIMPLE) and Momentum Interpolation (MI) on the collocated grids. Free surface of deposited PLA/GNPs nanocomposite filament and its streamlines are shown in Figure 3a,b.

## 3. Results and Discussions

### 3.1. Simulation Results and Analysis

#### 3.1.1. The Temperature Field and the Viscosity Field

**Figure 4:**
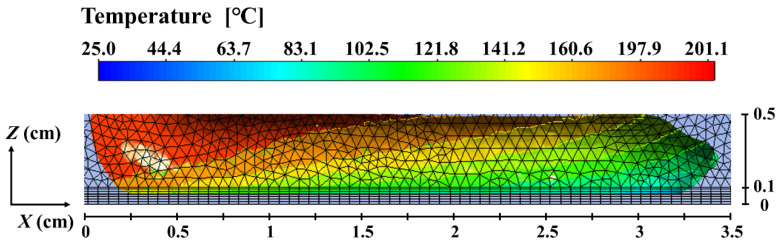
Surface temperature distribution of the deposited nanocomposite filament.

Figure 4 shows the surface temperature distribution of the deposited nanocomposite filament along its spreading direction. The cooling down of the bottom right filament and the highest temperature region in the top left corner of the just deposited filament can be observed clearly. The temperature gradient indicates that heat transfer occurs between the deposited nanocomposite filament and its surroundings during the deposition process. The convective heat transfer between the deposited nanocomposite melt and the ambient air as well as the heat conduction between hot nanocomposite filament and the substrate result in the rapid temperature drop on the front surface and the bottom right of the filament. 

**Figure 5:**
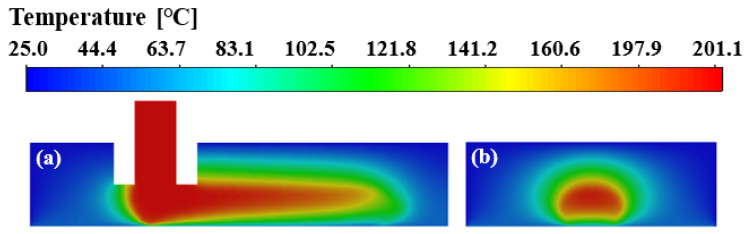
Temperature field in (**a**) the *x–z* plane, and (**b**) the cross-section at *x* = 0.6 mm.

The temperature depends on the heat transfer. Inside the printing chamber, the heat is added by the deposited nanocomposite and transferred away from the deposited hot filament to the surrounding cold air and substrate by heat conduction and thermal convection. To better study the effect of heat transfer on temperature of the deposited filament and surrounding air, the temperature field in the computational domain was examined. Figure 5a,b show the temperature fields of the deposited nanocomposite melt in the x–z plane and the cross-section at x = 0.6 mm, respectively. It can be clearly seen that the ambient air around the deposited filament is heated and its temperature increases significantly, forming a thermal effect zone consistent with the shape of the deposited filament. Due to the relatively low thermal diffusion rate compared to the nozzle velocity, it takes some time for the heat to fully diffuse to the surrounding area when the nanocomposite is deposited on the substrate. Therefore, when the temperature at the front end of the deposited filament almost drops to the solidification temperature, there is still a high temperature in the inner central region.

**Figure 6:**
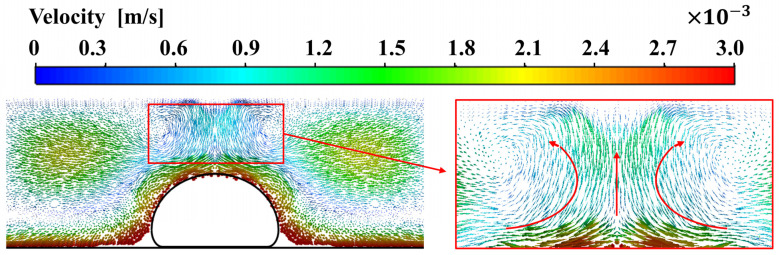
The velocity field induced by heat convection effect in the air around the filament.

Due to the movement of the deposited filament and the buoyancy caused by the temperature gradient, there is bound to be heat convection effect in the air around the filament, as shown in Figure 6. It can be seen that the hot air rises near the top of the hot filament, while the cold air in the distance flows down from the top of the domain, forming vortices in the relative motion path of hot and cold air on both sides of the deposited filament.

**Figure 7:**
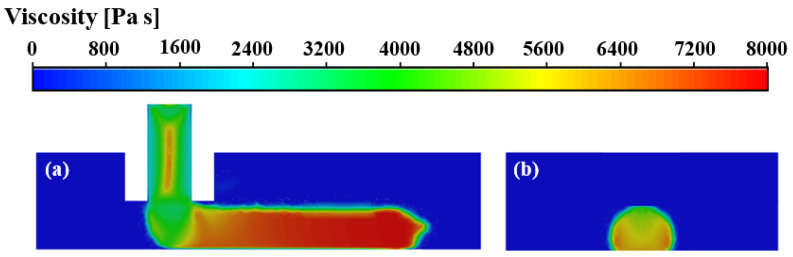
Viscosity fields of the deposited nanocomposite filament in (**a**) the *x–z* plane and (**b**) the cross-section at *x* = 0.6 mm.

The viscosity fields of the deposited nanocomposite filament are presented in Figure 7. The viscosity depends on the temperature and shear rate. It is clearly observed that the viscosity variation in the deposited filament closely corresponds to the temperature distribution, indicating that the viscosity is sensitive to the temperature. The viscosity value reaches its maximum at the bottom right of the front end of the deposited filament, and the bottom is larger than the top in the horizontal direction, as shown in Figure 7a. The viscosity is much lower in the filament adjacent to the nozzle outlet. The result indicates that there is a lower temperature and a higher shear rate at the bottom right of the front end of the deposited filament than that at the top back end. In the lateral direction, it is observed that the viscosity at the bottom and side wall of the filament is higher than that of the center and top, as shown in Figure 7b. It reveals that there is more heat transferred away from the deposited hot filament to the substrate than to the surrounding cold air. Meanwhile, the thermal convection on both sides of the deposited filament intensifies the heat transfer between the side wall of the filament and the surrounding air, resulting in temperatures at the bottom and side walls of the deposited filament being lower than that at the center and top.

**Figure 8:**
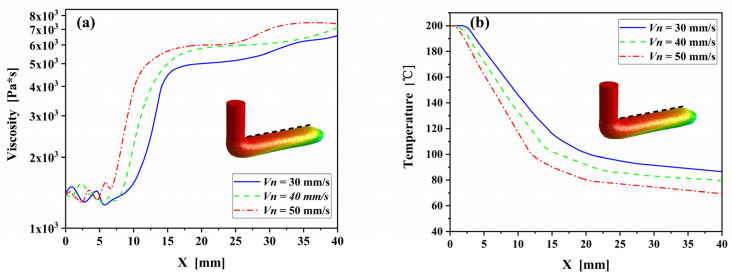
Temperature changes (**a**) and viscosity changes (**b**) along upper surface of PLA/GNPs nanocomposite melt at nozzle temperature of 200 °C.

To examine the role of the process parameters on temperature and viscosity of the deposited filament, the changes of temperature and viscosity along the upper surface of the deposited filament were extracted from the numerical results, as shown in Figure 8. As confirmed in the previous section, the temperature decreases and the viscosity increases along the deposition direction, intensely in the initial stage while slowly in the end section. With the increases of nozzle velocity, the sharp change in temperature and viscosity decreases from x = 17 mm with nozzle velocity of 30 mm/s to x = 12 mm with nozzle velocity of 50 mm/s. It indicates that the increase of nozzle velocity is favorable to the heat transfer between the fuse and its surroundings, accelerating the solidification of the filament.

#### 3.1.2. Evolution of the Cross-Section Morphology of the Deposited Filament

**Figure 9:**
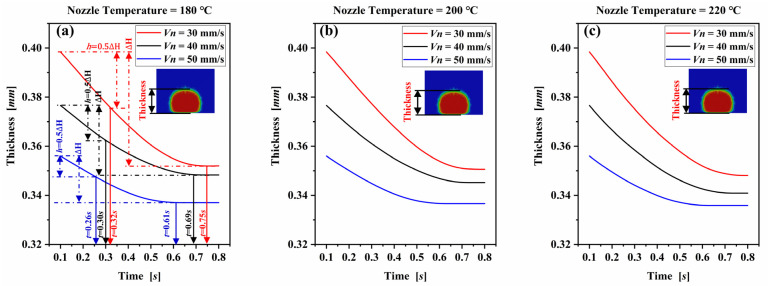
Evolution of the thickness of the deposited filament cross-section with time at different nozzle velocities in simulations: (**a**) nozzle temperature = 180 °C, (**b**) nozzle temperature = 200 °C, and (**c**) nozzle temperature = 220 °C.

To investigate the evolution of the deposited filament cross-section during solidification, the morphology of the cross-section was analyzed qualitatively, and its variation curve with time was plotted in Figure 9.

Nozzle velocity ($V_{n}$) and nozzle temperature ($T_{n}$) are the two key control variables affecting the dimension of the deposited filament. In order to study the influence of the two key control variables on the dimension of the deposited filament, the thickness and the width of the deposited filament versus time at different nozzle velocities and temperatures were calculated. Control variable method were adopted in the simulation, where the nozzle velocity was varied and the nozzle temperature was same. For the nozzle velocity study, the nanocomposite filament was deposited at three different nozzle velocities of 30 mm/s, 40 mm/s, and 50 mm/s with a same extrusion volumetric flux of 5.024 ${\mathrm{mm}}^{3}/s$. The evolution of the thickness of the extruded filament cross-section with time at different nozzle velocities and temperatures in simulations are presented in Figure 9. As can be seen from Figure 9a, during the deposition process, the thickness of the extruded filament gradually decreases with the extruded nanocomposite melt spreads on the substrate. The increase of the nozzle velocity reduces the thickness of the extruded filament. The thickness decreases from the initial deposition thickness of 0.398 mm to the final solidification thickness of 0.348 mm at nozzle velocity of 30 mm/s, with the thickness deformation rate of 12.6%. When the nozzle velocity increases to 40 mm/s and 50 mm/s, the thickness of the deposited nanocomposite melt decreases from the initial thickness of 0.377 mm and 0.356 mm to the solidification thickness of 0.341 mm and 0.336 mm, and the corresponding thickness deformation rates are reduced to 9.5% and 5.6%. The results indicate that there is a smaller thickness of the deposited nanocomposite filament and a smaller thickness deformation rate at a higher nozzle velocity condition. There are two reasons behind this phenomenon: (1) the thickness of the deposited filament is inversely proportional to the nozzle speed at a fixed extrusion volumetric flux condition. The higher the nozzle velocity, the smaller the initial thickness of the deposited filament. With the spreading and solidifying of the extruded nanocomposite melt on the substrate, there is a smaller solidification thickness of the deposited filament; and (2) heat transfer plays an important role in the solidification of the extruded nanocomposite melt. The coefficient of convection heat transfer between the deposited hot nanocomposite melt and ambient air increases with the decreasing the initial thickness of the deposited nanocomposite filament, which accelerates the solidification rate of the deposited nanocomposite and shortens the solidification time and thickness deformation of the deposited filament.

The effect of heat transfer on deposition thickness deformation can be confirmed from the thickness versus time curves. When the nozzle velocity is 30 mm/s, the thickness deformation of deposited filament reaches 50% (0.5ΔH) in 0.32 s, while the total deformation time is 0.75 s. It indicates that the thickness deformation rate decreases with the gradual decrease of the spreading temperature of the nanocomposite melt on the substrate. The decrease of thickness deformation rate versus time can be ascribed to the decrease of the temperature and the increase of the extruded nanocomposite melt viscosity caused by the heat transfer during the deposition process. In the initial deposition stage, the extruded nanocomposite melt has higher temperature and lower viscosity, resulting in greater deformation capacity. With the convection heat transfer between the deposited nanocomposite melt and the ambient air, as well as the heat conduction between hot nanocomposite filament and the substrate in the deposition process, the temperature of the extruded nanocomposite melt gradually decreases. This leads to the increase of nanocomposite melt viscosity and difficult deformation. With the time t = 0.75 s, the deposited filament does not further decrease in thickness with time, which indicates that the temperature of the extruded nanocomposite melt is lower than the glass transition temperature due to the convection heat transfer and heat conduction. At this time, the deposited nanocomposite filament has solidified and the corresponding solidification time is 0.75 s. For the higher nozzle velocities of 40 mm/s and 50 mm/s, the time when the thickness deformation reaches 50% (0.5 ΔH) of total deformation is 0.30 s and 0.26 s, their total deformation times are 0.69 s and 0.61 s. It reveals that, compared to the lower nozzle velocity, high nozzle velocity printing condition intensifies the heat transfer between the deposited nanocomposite filament and ambient air or substrate, then accelerates the solidification rate and shortens the solidification time. Figure 9b,c show the thickness of the extruded filament versus time at nozzle temperature of 200 °C and 220 °C. The results show that the deposited nanocomposite filament has a smaller solidification time, a bigger thickness and a smaller thickness deformation rate at a higher nozzle velocity condition can be obtained.

**Figure 10:**
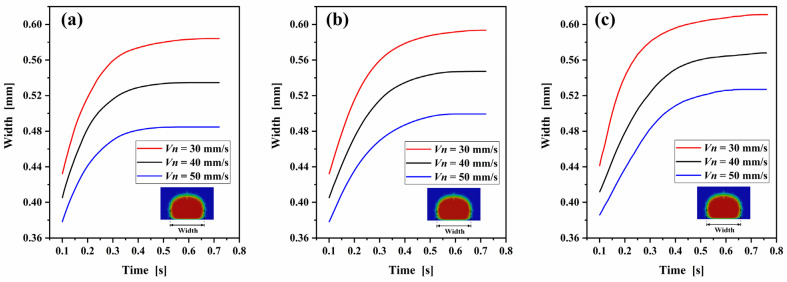
Evolution of the width of the extruded filament cross-section with time in simulations of different nozzle velocity: (**a**) nozzle temperature = 180 °C, (**b**) nozzle temperature = 200 °C, and (**c**) nozzle temperature = 220 °C.

The influence of the nozzle velocity on the width of the deposited filament is presented in Figure 10. Figure 10a shows the width of the extruded filament cross-section versus time at nozzle temperature of 180 °C. It can be seen that increasing the nozzle velocity reduces the width of the extruded filament. The width of the extruded nanocomposite filament increases from the initial deposition width of 0.432 mm to the final solidification width of 0.584 mm at a nozzle velocity of 30 mm/s, with the solidification time of 0.65 s and width deformation rate of 35.2%. When the nozzle velocity increases to 40 mm/s and 50 mm/s, the solidification widths reduce to 0.535 mm and 0.485 mm, and the corresponding width deformation rates are 32.1%, 28.3%. The results indicate that increasing the nozzle velocity leads to a smaller width and a smaller width deformation rate of the deposited nanocomposite filament. Figure 10b,c show the width of the extruded filament versus time at nozzle temperature of 200 °C and 220 °C, and the results confirm the above conclusion obtained again.

**Figure 11:**
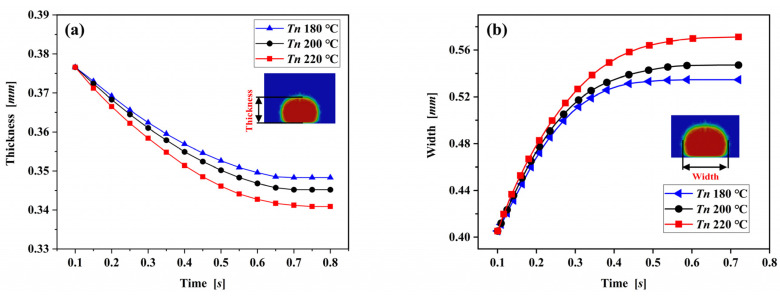
(**a**) Thickness and (**b**) width of the extruded filament versus time for three different nozzle temperatures with nozzle velocity of 40 mm/s.

Changes in nozzle temperature may not affect the deposition process as intuitively as changes of nozzle velocity, but it strongly affects the heat transfer between the deposited filament and the substrate and ambient air, thus determining the dimension of the deposited filament. To study of the role of nozzle temperature on the dimension of the extruded filament, the comparation of the effect of nozzle temperature on the thickness and width of the extruded filament were simulated. Figure 11 shows the thickness and the width of the extruded filament versus time under three different nozzle temperatures at nozzle velocity of 40 mm/s. It can be seen that the increases of the nozzle temperature prolong the solidification time and decreases the thickness of the deposited nanocomposite filament. On the contrary, increasing the nozzle temperature increases the width of the deposited nanocomposite filament. Calculations indicate that the increase of nozzle temperature decreases the viscosity and increase the flow capacity of the deposited PLA/GNP melt. Meanwhile, higher nozzle temperature intensifies the heat transfer and prolongs the solidification time, finally increasing the thickness and decreasing the width of the deposited nanocomposite filament. The decrease of nozzle velocity and the increase of nozzle temperature promote the flow of the deposited nanocomposite melt and prolong the solidification time, resulting in a smaller thickness with larger width. The results fully prove that the nozzle temperature is also a sensitive variable to the dimension of the extruded filament.

### 3.2. Experimental Validation

#### 3.2.1. Validation of the Influence of Printing Parameters on the Morphology and Dimension of the Deposited Filament

**Figure 12:**
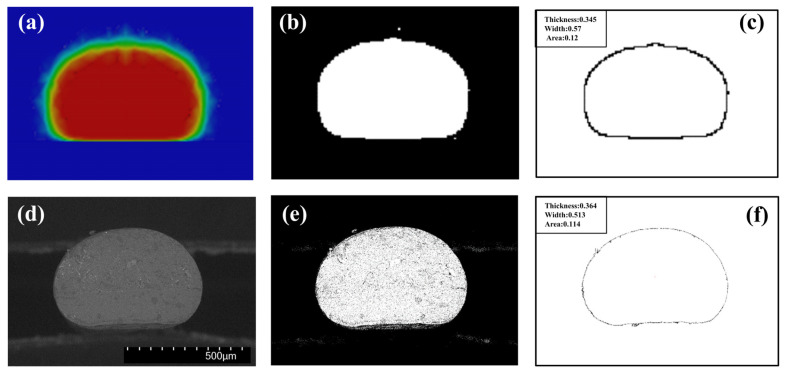
The contours and dimensions of the filament cross-sections from the computational and experimental morphologies: the computational (**a**) and the experimental SEM (**d**) morphologies; (**b**,**e**) are the binary black-and-white image calculated by threshold slicing algorithm; and (**c**,**f**) are the contours with thickness, width, and the mean area measured by ImageJ.

In this part, morphology and dimension of the deposited nanocomposite filaments predicted by numerical simulations were compared with an experimental procedure under different printing parameters. Nine PLA/GNPs nanocomposite filament specimens were extruded by CreatBot DE Printer (Henan SuWei Electronic Technology Co., Ltd., Henan, China). After printing, the nanocomposite filaments were cut perpendicular to the printing direction and the cross-section morphologies were observed by the scanning electron microscope (SEM). The computational and experimental contours of the filament cross-sections were extracted from numerical simulation results and SEM morphologies analyzed by using an open-source image processing software, ImageJ. The thickness, width and the mean area of the filament cross-section were calculated by the threshold slicing algorithm. Examples of the contours and the dimension of the filament cross-section from the computational and experimental morphologies are shown in Figure 12. The computational morphology (Figure 12a) and the experimental SEM morphology (Figure 12d) are processed to a binary black-and-white image with only black and white colors by appropriate thresholding, as shown in Figure 12b,e. The contours of the filament cross-section were extracted and the thickness, width, and the mean area were measured from the binary black-and-white image, as shown in Figure 12c,f.

**Figure 13:**
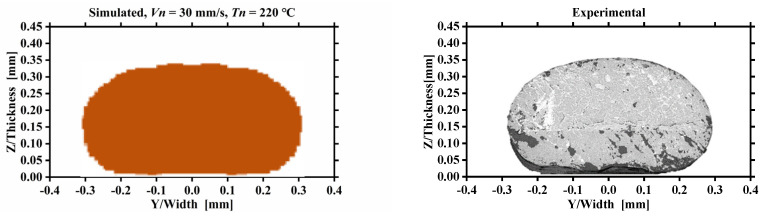

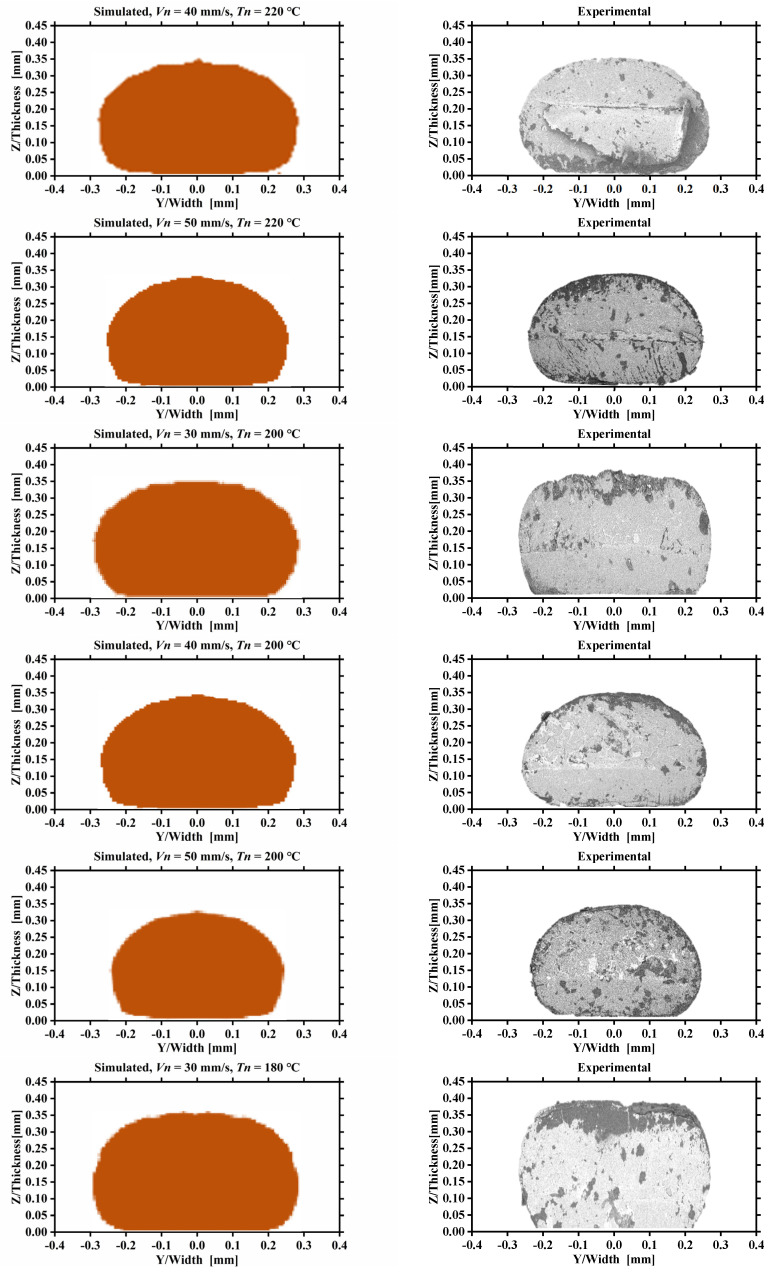

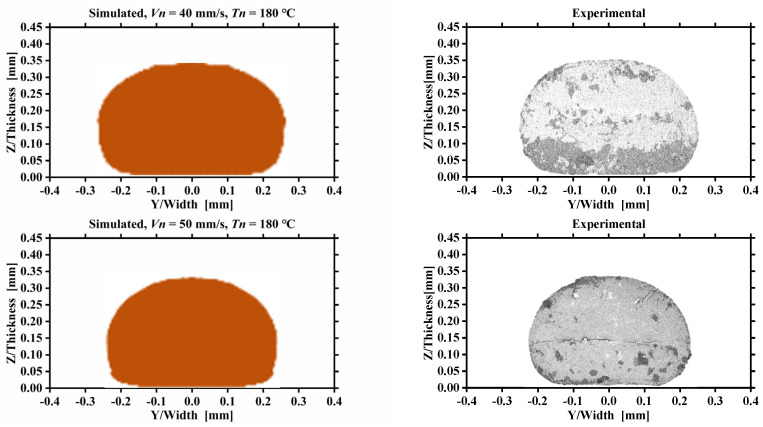
Qualitative comparison of the simulated (**left**) and experimental (**right**) cross-section morphologies of deposited nanocomposite filaments extruded with different printing parameters.

Figure 13 represents the comparison of the simulated (left column) cross-section morphologies of the deposited nanocomposite filament with the experimental observations (right column), the nozzle velocity and temperature are specified in Table 1. As exhibited in all diagrams, the simulated cross-section morphologies are basically consistent with those measured in the experiments, which confirms the accuracy of the numerical simulations. The cross-section morphologies of the deposited nanocomposite filament sensitively depend on both nozzle velocity and temperature. With the decrease of nozzle velocity $V_{n}$ and the increase of nozzle temperature $T_{n}$, the cross-section morphologies change from a circular to a long oval with larger top curvature radius. When the nozzle velocity is high ($V_{n}$ = 50 mm/s) and nozzle temperature is low ($T_{n}$ = 180 °C), there is less hot nanocomposite extruded onto per distance of the filament, and the deposited filament will not be squeezed by the nozzle after leaving it, forming an approximate circular cross-section morphology. When the nozzle velocity decreases to 30 mm/s, because the gap distance is fixed and the extrusion volumetric flux inside the nozzle is constant, there is too much hot nanocomposite extruded onto per distance of the filament, resulting in side flow of the nanocomposite filament. Increasing the nozzle temperature enhances the side flow under the condition of low nozzle velocity ($V_{n}$ = 30 mm/s). There is a visible dent on the middle of the top filament cross-section with $V_{n}$ = 30 mm/s as observed by SEM. This process condition results in a decrease in printing accuracy and should be avoided. Due to the mass conservation, the decrease of nozzle velocity and the increase of nozzle temperature both increase the side flow of deposited filament, which flattens the cross-section morphologies of deposited filaments.

**Figure 14:**
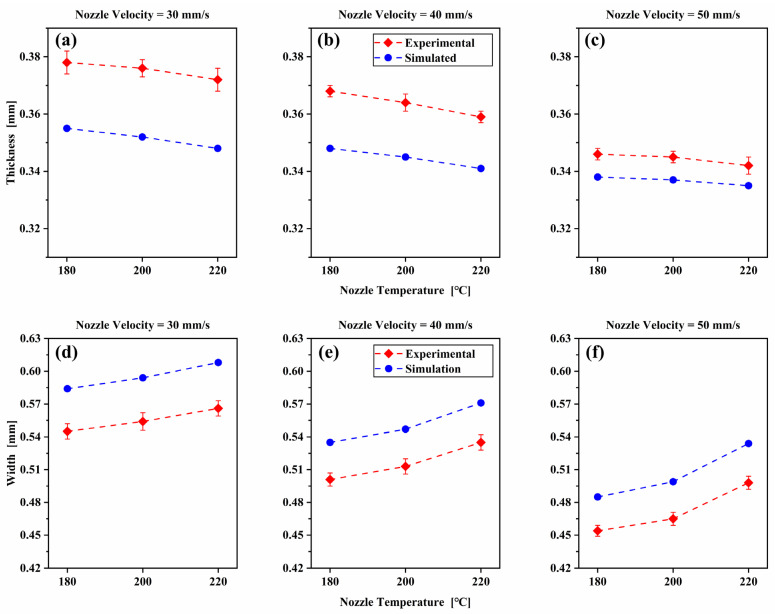
Comparison of the simulated and experimental thickness (**a**–**c**) and width (**d**–**f**) of the deposited nanocomposite filament cross-section for different printing parameters.

In order to verify the influence of printing parameters on the dimension of deposited filament, the thickness and width of deposited filament cross-section measured in experiments and calculated in simulations are quantitatively compared, as shown in Figure 14. The effects of the processing parameters on the variation trend of the width and thickness observed in experiments are consistent with the predictions from simulations. Seen from Figure 14a–c, it can be noted that the filament thickness decreases gradually with the increase of nozzle velocity $V_{n}$ and nozzle temperature $T_{n}$. The maximum reduction in thickness is 10.5%. Compared with the experimental results, all the simulated filament cross-section thicknesses are under-estimated. Contrary to the hypothesis in the literature that the deposited filament thickness is equal to the gap distance, the numerical and experimental thickness of the filament varies between 83.8% and 94.5% of the gap distance, within the scope of the printing parameter we studied. It can be observed that there is a large thickness deviation between simulation and experiment results at high nozzle temperature. This is due to the deposited hot nanocomposite attached to the substrate and the cap of the moving nozzle, when the nanocomposite is extruded onto the substrate, squeeze deformation occurs in the gap between the substrate and the nozzle. After cooling and solidification, the filament can yield shrinkage.

Contrary to the thickness trend, the width increases with the increase of nozzle temperature $T_{n}$ and decreases with the decrease of nozzle velocity $V_{n}$. The maximum increase in width of the deposited filament is 24.7%, as present in Figure 14d–f. Similar width trend is observed from numerical simulation and discussed in the previous section. Compared with the experimental results, the cross-section width of the simulated filament is slightly higher. The under-estimated thickness and over-estimated width indicate that the numerical model gives the nanocomposite material too much fluidity. The deviation can be compensated by reducing the material viscosity parameter.

**Figure 15:**
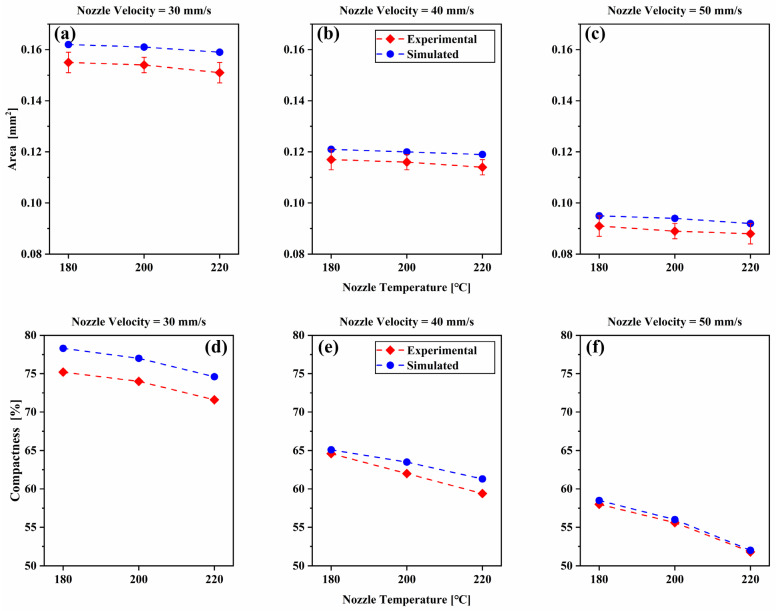
Comparison of the simulated and experimental area (**a**–**c**) and compactness (**d**–**f**) of the deposited nanocomposite filament cross-section for different printing parameters.

Comparisons of the area and the compactness (expressed by the ratio of the filament cross-section area and the product of the thickness and width) of the filament cross-section are presented in Figure 15. The filament cross-section area increases dramatically with the decrease of nozzle velocity $V_{n}$. For a given nozzle velocity, the filament cross-section area decreases slightly with the increase of nozzle temperature. It reveals that, compared with nozzle temperature, nozzle velocity has a relatively more significant effect on the filament cross-section area. Under the condition of high nozzle velocity, the filament cross-section area decreases slightly, which is due to the high volume expansion rate of the deposited nanocomposite at high temperatures. The compactness decreases with the increase of nozzle velocity $V_{n}$ and nozzle temperature $T_{n}$. It can also be seen that of the filament cross-section compactness under the condition of high nozzle velocity obtained by simulation is more accurate.

#### 3.2.2. Effect of Printing Parameters on the Mechanical Property of the Printed PLA/GNPs Nanocomposite

In this section, tensile strength and dimensional accuracy of the printed PLA/GNPs nanocomposite part are evaluated under different printing parameters. Nine tensile specimens were designed according to ASTM D638 standard [48] and printed on a CreatBot DE Printer. Except for nozzle velocity and nozzle temperature, the other printing parameters such as build orientation, raster angle, infill density, and layer thickness are kept fixed for all specimens, as shown in Table 3.

**Figure 16:**
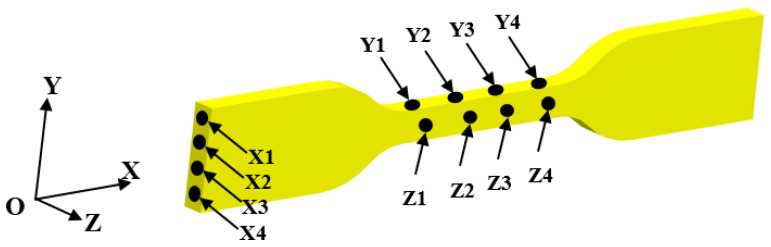
The arrangement of the measuring points of FDM printed specimens.

The uniaxial tensile tests of the specimens were performed on an electronic universal testing machine (CTM 8010, XieQiang Instrument manufacturing, Shanghai) equipped with 10 KN load cell. The tests were conducted at a fixed loading rate of 5 mm/min according to ASTM standard. The dimensional accuracy of printed part can be characterized by calculating the standard deviation of the sample size [49]. The dimensional data are obtained by measuring the surface dimensions in X-dimension, Y-dimension, and Z-dimension of the specimens with a vernier caliper. Four points on each surface of the specimens were selected to measure the dimensions and calculate the standard deviation. The arrangement of the measuring points is shown in Figure 16, and the corresponding dimensional data is shown in Table 4.

**Figure 17:**
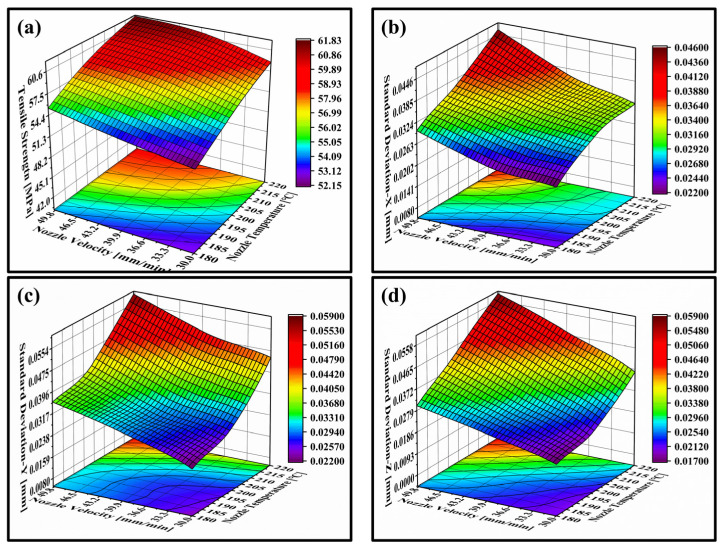
3D response surface plots showing the interaction effects of nozzle temperature and nozzle velocity on (**a**) the tensile strength; (**b**) standard deviation in X-dimension; (**c**) standard deviation in Y-dimension; (**c**) standard deviation in Z-dimension; and (**d**) standard deviation in Z-dimension of the FDM printed part.

The tensile strength and the dimensional standard deviation of the specimens under different process parameters are reported in Table 5. In order to analyze the effect of process parameters on the tensile strength and dimensional accuracy of FDM printed PLA/GNPs nanocomposite more intuitively and conveniently, the parameter-effects 3D response surface plots were established according to results in Table 5, as shown in Figure 17.

As seen from Figure 17a, the specimens at the upper-left corner of the plot have excellent mechanical properties and tensile strength greater than 61.8 MPa, while in the bottom-right corner of plot, the specimens exhibit low mechanical properties and the tensile strength less than 52.1 MPa. In the high-strength printing region, the corresponding nozzle temperature is greater than 205 °C, nozzle velocity is greater than 38 mm/s. This result indicates that the increase of the nozzle velocity and nozzle temperature increases the tensile strength of the printed specimens, and the maximum tensile strength could be raised by 18.6%. The reason is mainly because the wettability and fluidity of extruded filaments are enhanced under the condition of higher nozzle temperature, which will improve the bonding area and bonding strength of adjacent filaments. In addition, the higher nozzle velocity is beneficial to shorten the curing time of the filament, reduce the forming time interval of the successive layers, thus enhancing the interlayer and intralayer bonding strength and tensile strength of FDM prints. In the contour plot, it can also be seen that the selection window of nozzle velocity is larger than that of nozzle temperature to achieve higher tensile strength, which indicates that nozzle temperature has a more sensitive and significant effect of on the strength of FDM printed sample than nozzle velocity.

Figure 17b–d represent the 3D response surface plots of the relationships between the process parameters and the dimensional accuracy (represented by standard deviation) of FDM printed specimens in X, Y, and Z dimensions. It can be seen from these plots that both nozzle velocity and nozzle temperature have a significant impact on the dimensional accuracy of FDM printed parts. The decrease of the nozzle velocity and nozzle temperature can reduce the dimensional standard deviation of FDM printed specimens and improve the printing accuracy. The dimensional standard deviation of printed specimens decreased by 52.2%, 62.7%, and 68.3% in the X, Y, and Z direction under the lower nozzle velocity and temperature condition. According to Figure 17, it is convenient to predict the tensile strength and dimensional accuracy of FDM parts in experiments and to select reasonably printing parameters to obtain an ideal tensile strength and dimensional accuracy. In this study, 32–46 mm/s of nozzle velocity and 202–212 °C of nozzle temperature were the optimal process parameters for FDM printing PLA/GNPs nanocomposite to obtain the ideal tensile strength and dimensional accuracy.

## 4. Conclusions

In this study, we propose a numerical model to simulate the FDM 3D deposition process of PLA/GNPs nanocomposite in the process by computation fluid dynamics (CFD) paradigm. The numerical simulation of the FDM process involves the flow of molten material and free surface movements, an appropriate rheological model was used to represent the viscosity of printing material and the heat transfer between the filament and the surrounding air. The numerical model allows us to predict the cross-sectional morphology and dimension of FDM filament under different process parameters, and investigate the effect of process parameters on mechanical property of FDM printed samples. The proposed numerical model fully simulated the deposition process of the fused filament and accurately calculated the size and shape of the solidified filament. Both the numerical simulation and experiment results show that the cross-section of the deposited PLA/GNPs nanocomposite filament is almost cylindrical at low nozzle temperature and high nozzle velocity, while a flat cuboid with rounded edges under the condition of high nozzle temperature and low nozzle velocity. The decrease in thickness and the increase in width of the deposited filament caused by the increase of nozzle temperature and decrease of nozzle velocity, the width of the deposited filament decreased by10.5% and the thickness increased by 24.7%. In addition, the experimental results indicate that with the nozzle temperature increased from 180 °C to 220 °C and the nozzle velocity increase from 30 mm/s to 50 mm/s, the tensile of the printed PLA/GNPs specimen increased by 18.6%. Moreover, the nozzle temperature has a more sensitive and significant effect on the strength of printed PLA/GNPs specimens than the nozzle speed. Both nozzle velocity and nozzle temperature have significant effects on the dimensional accuracy of the printed PLA/GNPs specimens. With the decrease of nozzle velocity and temperature, the printing accuracy of printed PLA/GNPs specimens improved. The dimensional standard deviation of printed specimens decreased by 52.2%, 62.7%, and 68.3% in the X, Y, and Z direction, respectively.

## Figures and Tables

**Table 1:** Summary of the printing parameters used in simulations.

Parameters	Symbol	Value	Unit
Nozzle velocity	$V_{n}$	30, 40, 50	mm/s
Nozzle temperature	$T_{n}$	180, 200, 220	°C
Air temperature	$T_{a}$	25	°C
Nozzle diameter	*D*	0.4	mm
Feed rate (average flux inside nozzle)	$Q_{fr}$	5.024	${\mathrm{mm}}^{3}/s$
Substrate temperature	$T_{s}$	25	°C
Gap distance	*g*	0.4	mm

**Table 2:** Properties of PLA/GNPs nanocomposite and air used in this study.

Parameters	Signal	Value
Density of PLA/GNPs nanocomposite (kg/m^3^)	$\rho_{p}$	1300
Viscosity of PLA/GNPs nanocomposite (Pas, range-function of T)	$\mu_{p}$	20–8000
Thermal conductivity of nanocomposite (W/mK)	$k_{p}$	0.195
Specific heat capacity of nanocomposite (J/kg K)	$c_{p,p}$	2000
Surface tension coefficient (kg/s^2^)	σ	0.04
Density of air (kg/m^3^)	$\rho_{a}$	0.9
Viscosity of the air (Pa s)	$\mu_{a}$	$2.3\times 10^{-5}$
Thermal conductivity of air (W/mK)	$k_{a}$	0.034
Specific heat capacity of air (J/kgK)	$c_{p,a}$	1000

**Table 3:** Process parameters of the FDM printed specimens.

Parameters	Value
Build orientation	Horizontal
Raster angle	45/−45
Infill density	100%
Layer thickness	0.2 mm

**Table 4:** Dimensional measurements of the specimens under different process parameters.

$V_{n}\;\mathrm{mm}/s$	$T_{n}$ °C	X-Dimension (mm)				Y-Dimension (mm)				Z-Dimension (mm)			
		X1	X2	X3	X4	Y1	Y2	Y3	Y4	Z1	Z2	Z3	Z4
30	180	115.14	115.18	115.12	115.14	6.18	6.24	6.22	6.20	3.96	3.92	3.96	3.94
30	200	115.16	115.14	115.18	115.22	6.24	6.28	6.20	6.24	3.88	3.94	3.9	3.92
30	220	115.24	115.18	115.22	115.16	6.24	6.32	6.20	6.28	3.94	3.88	3.9	3.84
40	180	115.16	115.18	115.12	115.18	6.20	6.26	6.22	6.18	3.88	3.92	3.94	3.94
40	200	115.18	115.14	115.22	115.14	6.32	6.24	6.28	6.24	3.86	3.94	3.88	3.86
40	220	115.26	115.18	115.20	115.16	6.28	6.34	6.20	6.28	3.82	3.94	3.84	3.90
50	180	115.22	115.18	115.14	115.16	6.20	6.28	6.26	6.20	3.92	3.86	3.94	3.90
50	200	115.16	115.24	115.18	115.14	6.34	6.28	6.34	6.24	3.82	3.86	3.94	3.86
50	220	115.24	115.26	115.28	115.16	6.36	6.30	6.22	6.22	3.78	3.92	3.92	3.84

**Table 5:** Tensile strengths and standard deviations of the sample dimensions.

$V_{n}\;\mathrm{mm}/s$	$T_{n}$ °C	Tensile Strength (MPa)	Standard Deviation in X-Dimension	Standard Deviation in Y-Dimension	Standard Deviation in Z-Dimension
30	180	52.15	0.022	0.022	0.014
30	200	54.96	0.030	0.028	0.024
30	220	57.82	0.032	0.045	0.037
40	180	54.62	0.024	0.030	0.017
40	200	57.34	0.033	0.033	0.029
40	220	60.23	0.037	0.050	0.046
50	180	56.34	0.030	0.036	0.029
50	200	59.13	0.037	0.042	0.041
50	220	61.83	0.046	0.059	0.051
